# Metastatic epithelioid sarcoma on the arm: presentation of a case report

**DOI:** 10.1093/jscr/rjaf733

**Published:** 2025-09-13

**Authors:** Jinxian Luo, Lei Yang

**Affiliations:** Department of Thyroid and Mammary Surgery, The Affiliated Guangdong Second Provincial General Hospital of Jinan University, Courtyard No. 466 Xingang Middle Road, Haizhu District, 510310, Guangzhou, China; Pingshan Hospital, Southern Medical University, Pingshan District Peoples’ Hospital of Shenzhen, Pingshan Street, 518118, Shenzhou, Guangdong, P.R. China

**Keywords:** diagnosis, ERG, metastatic, epithelioid sarcoma, case report

## Abstract

Epithelioid sarcoma (ES) is a highly malignant soft tissue tumor characterized by its tendency to distant metastasis, regional lymph node involvement, and recurrence. ES frequently manifests as a deep dermal mass in the distal extremities of young adults. The variety of pathological morphology makes it clinically misdiagnosed as a granuloma or rheumatoid nodule frequently. In this case, we report a new case of a 56-year-old female who was diagnosed with metastatic ES on her left upper arm. The diagnosis was confirmed by pathological biopsy and immunohistochemistry analysis, and metastatic after surgical excision. Therefore, we should implement biopsy and immunohistochemistry methods on patients to rule out rare ES.

## Introduction

Epithelioid sarcoma (ES) is a rare malignant soft tissue sarcoma, representing ˂1% of all soft tissue sarcomas. This name was originally proposed by Enzinger in the 20th century. The disease is more predominant among adolescents, with a higher occurrence in males than females [1]. Due to their unclear histogenesis, slow growth, and similarities in pathological manifestations with benign tumors, ES are often disregarded and misdiagnosed by clinicians [2]. In clinical practice, typical symptoms mainly present as painless nodules with ulcers and necrosis in the superficial area, as well as a high risk of distant metastasis and recurrence. ES is mainly categorized into distal type (especially extremities) and proximal type (head, neck, and trunk), based on its location [3]. Here we describe a middle-aged woman who presented with swelling on the left upper arm and left hand. After conducting a histopathological analysis, it was determined that she had ES in her left arm, accompanied by lymph node metastasis.

## Case report

A 56-year-old woman presented to the Department of Thyroid and Breast Surgery at her hospital with a painless swelling on her left upper arm for a 5-month history. The swelling was grew slowly and affected the normal function of her hand. Previously, there was no history of trauma. For the first time, the simple tumor excision was performed in April 2023. Meanwhile, we received a specimen for the pathological opinion, which indicated proximal-type ES on the left upper arm. Furthermore, the patient underwent drug chemotherapy (Epirubicin and Ifosfamide) in May and June 2023, respectively. In June 2023, there was a clinical recurrence of nodular lesions without significant changes to the skin, and an axillary mass appeared. The contrast-enhanced computed tomography of the left upper arm and palm showed multiple enlarged lymph node shadows on the left axilla.

**Figure 1:**
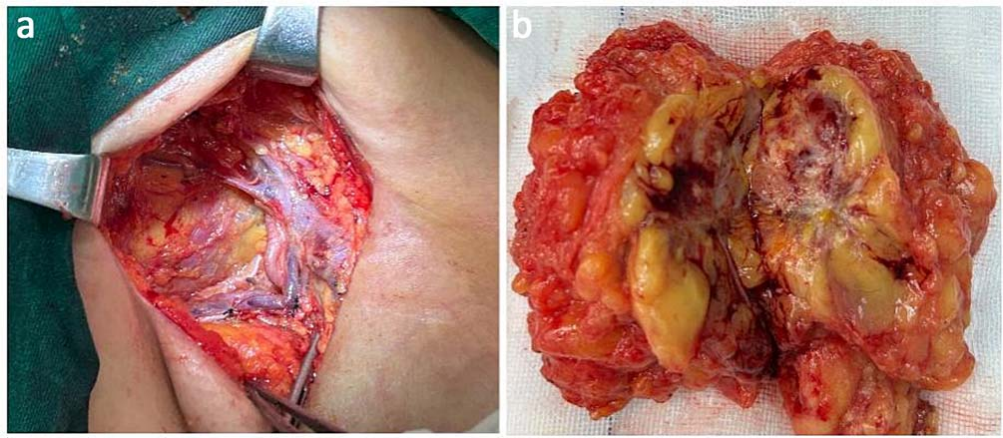
Presentation of the surgical image of tumor resection in the axillary region (a). Metastatic proximal-type ES presented as ill-defined multinodular masses in the muscle of the axillary region during intraoperative (b).

**Figure 2:**
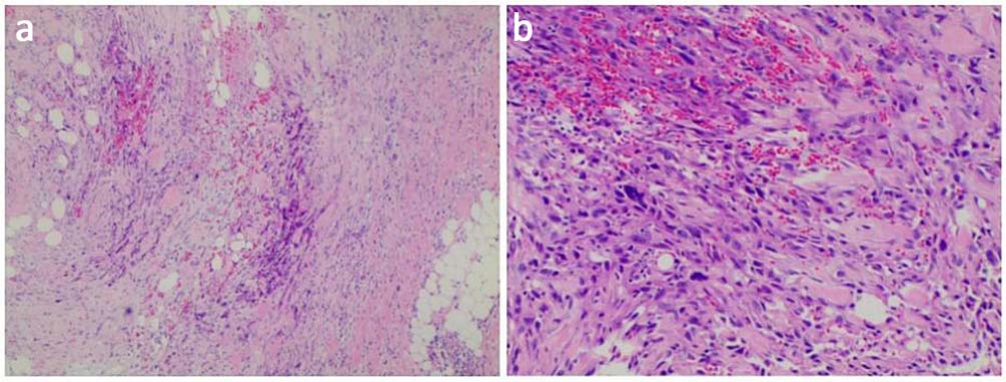
HE stained sections showed cellular tumor and local tumor necrosis (a); the epithelioid cells arranged in a cord shape, eosinophilic cytoplasm, and scattered rhabdoid cells (b).

**Figure 3:**
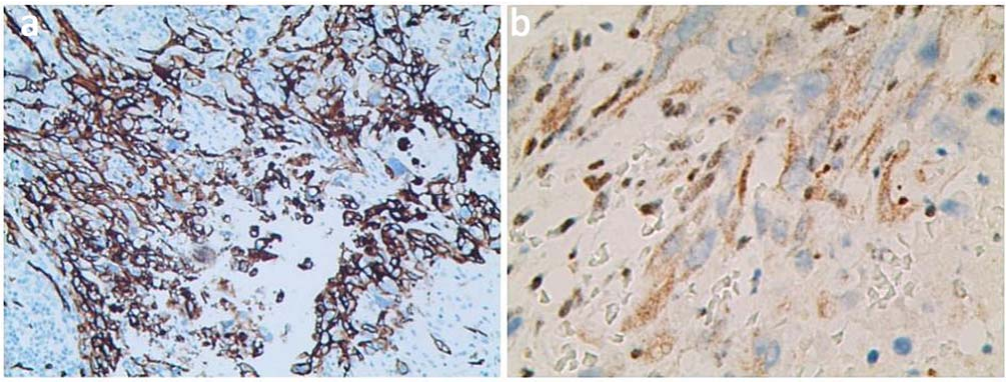
Immunohistochemical stained for CD34 (a) and INI1 (b).

The axillary lymph node resection was performed completely under general anesthesia. Subsequently, lymphadenopathy with a size of 2.6 × 2.2 cm was discovered. Upon examination, the lesions appeared as ill-defined, multicellular whitish masses with evidence of hemorrhage and necrosis (Fig. 1). The histopathological diagnosis revealed the presence of focal spindle cells and epithelioid cells, indicating metastatic ES from the tumor on the left (Fig. 2). Immunohistochemical staining results demonstrated the positivity for tumor markers CK/pan, CD34, ERG, P53, while the negativity for INI1 and CD31 (Fig. 3). Consequently, the diagnosis of metastatic ES was further confirmed. After the operation, the patient successfully recovered and was safely discharged.

## Discussion

Clinically, ES is a rare epithelioid soft tissue tumor characterized by local recurrence and distant metastases. However, the histological origin remains unclear [4]. ES often presents in the distal extremities, with a predilection for the palms, wrists, and forearms. It also exhibits a susceptibility to early metastasis [5]. Simultaneously, this rare disease may be misdiagnosed, leading to delay in treatment and diagnosis for patients [6]. In this case, we present a rare manifestation of ES on the upper arm.

Compare with other types of soft tissue sarcoma, ES has a relatively high rate of metastasis and recurrence [7]. Previous studies have indicated that the lungs and regional lymph nodes are the common sites of metastasis for ES [8]. Additionally, lymph node metastasis is often a risk factor for poor prognosis in ES, although the patient’s age, sex, site, tumor size and depth, and mitotic rates are also important prognostic indicators [9]. The 5-year survival rate of female patients (80%) is significantly higher than that of male patients (40%), suggesting a better prognosis for females [10]. Furthermore, elderly patients with multiple local lesions, tumor invasion, high mitotic rate, tumor necrosis, hemorrhage, and high lymphocytic infiltrate tend to have poor survival outcomes due to distant metastasis [11]. In the present case, a female patient with ES accompanied by axillary lymph node metastasis was analyzed to have good prognostic efficacy.

Histology serves as a diagnostic indicator of ES, with histological characteristics mainly including epithelioid cells and spindle-shaped cells, mitotic figures, cytologic atypia, eosinophilic, and vesicular nuclei [12]. Immunohistochemical detection helps differentiate ES from epithelioid hemangioma, synovial sarcoma, squamous cell carcinoma, malignant rhabdoid tumor, and malignant melanoma [13]. ES shows characteristic immune reactivity to epithelial markers and vimentin, such as CD34, CK/pan, and EMA. CD34 expression in PES lesions is ⁓50%, often used as a diagnostic marker in vimentin negative patients. Moreover, as an endothelial marker of CD34 shows positive reactivity conducive to a diagnosis of PES.

INI1 is a component of the core protein subunits in the ATP-dependent chromatin-remodeling complex SWI/SNF, and its expression regulation is a significant epigenetic mechanism [14]. Loss of the INI1 gene leads to uncontrolled gene expression. Hornick *et al.* [15] reported that 90% of ES patients have a deletion of the INI1 gene. Therefore, the loss of INI1 has an essential reference value for the diagnosis of ES diseases. ERG belongs to the specific erythroblast transformation of transcription factors, which regulate angiogenesis and endothelial cell apoptosis and playing a crucial role in blood development [16]. Some researchers reported that 41% of ERG-positive patients had expression in INI1-deficient ES, including 13 of 24 conventional types and 5 of 20 cases with proximal-type of ES. These results suggested that ERG expression may support the proximal-type epithelioid sarcoma (PES) differentiation [17]. Thus, in our case, the ES with the positive expression of ERG was further confirmed.

## Conclusion

ES is a rare and highly invasive malignant tumor that poses a serious challenge to clinicians due to its poor prognosis. Due to the frequent misdiagnosis of ES as another malignant or benign tumor disease, its correct diagnosis is crucial for us. Therefore, we usually use pathological biopsy, tumor markers, and immunohistochemical examination to identify the disease. Surgical excision, chemotherapy, and radiation therapy are often used as treatment methods for ES, but ES still maintains a high recurrence rate. With in-depth research on the pathogenesis of ES, we anticipate the emergence of innovative therapeutic techniques in the future.
